# The Economics of Mitigating Flexible Hydropower: A Systematic Review

**DOI:** 10.1007/s00267-026-02488-z

**Published:** 2026-04-30

**Authors:** Nusrat Jahan Bipa, Giuseppe Roberto Pisaturo, Terese E. Venus

**Affiliations:** 1 Free University of Bozen-Bolzano, Faculty of Engineering, via Bruno Buozzi 1, Bolzano, Italy; 2 Sustainable Development and Climate Change, University School for Advanced Studies IUSS, Pavia, Italy; 3 Bioeconomy Economics Research Group, University of Passau, Passau, Germany

**Keywords:** Hydropeaking mitigation, Hydropower, Environmental externalities, Energy market design, Renewable energy transition

## Abstract

Flexible hydropower is essential for integrating variable renewables, yet its short-term flow fluctuations (hydropeaking) can result in negative ecological impacts and, when mitigated, financial losses for operators. As balancing these impacts with energy system flexibility represents a key policy challenge for sustainable energy transitions, we conduct a systematic review of the economic dimensions of hydropeaking mitigation using the PRISMA methodology. We identify 35 studies and find that operational mitigation measures (e.g., ramping restrictions, minimum flows) can be effective in reducing ecological stress but may also reduce revenue by 1–8%, depending on market conditions and the perceived severity of the constraints. Structural mitigation measures, such as re-regulation reservoirs and compensation basins, lead to better ecological effectiveness by buffering flow variability, while also maintaining long-term economic feasibility, particularly when designed for multipurpose use. Emerging new hybrid mitigation measures, especially hydro-battery energy storage systems, exhibit potential to balance ecological objectives with system flexibility and market profitability. While some mitigation measures reduce hydropower revenues, several studies indicate that the associated environmental improvements can generate broader economic benefits for society, potentially resulting in net welfare gains. Additionally, the review highlights recent methodological advances used to assess economic trade-offs in hydropeaking mitigation contexts. Overall, environmental externalities associated with hydropeaking are often not fully internalized in market outcomes, with implications for hydropower regulation and market design. Policy frameworks such as environmental flow regulations, compensation mechanisms, and targeted support for mitigation infrastructure may help internalize environmental externalities and incentivize hydropower operation that balances system flexibility with river ecosystem protection.

## Introduction

Hydropower is a clean and flexible source of renewable energy that supports the integration of variable generation into modern power systems (Halleraker et al., 2022; Hayes et al., 2023; Venus et al., 2020; Venus et al., 2025). By adjusting electricity production to match periods of high demand and price, hydropower plants help stabilize the grid and maximize profitability (Olivares and Lund, 2012). This operational flexibility, a phenomenon known as hydropeaking, can lead to rapid and frequent fluctuations in river flows. In turn, these abrupt flow changes can impose substantial environmental and social costs, including ecosystem degradation, biodiversity loss, and increased risks to riverine safety and recreation (Bipa et al., 2024).

A range of mitigation strategies has been developed to address the environmental and social impacts of hydropeaking. These include operational restrictions, retention basins, bypass channels, and riverbed restoration. Each approach involves complex trade-offs: while measures can reduce hydropower revenues, incur infrastructure costs, and lower system flexibility, they can also generate long-term environmental and social benefits such as improved biodiversity, increased recreational opportunities and regulatory compliance (Person et al., 2014; Niu and Casas-Mulet et al., 2016; Ruokamo et al., 2024; Insley, 2013). Assessing these trade-offs is further complicated given that many benefits are also difficult to quantify in monetary terms, particularly cultural and supporting ecosystem services (Bruder et al., 2016; Rodríguez et al., 2006; Venus and Sauer, 2022).

Although research on the ecological impacts of hydropeaking and the effectiveness of associated mitigation strategies has advanced significantly (Vanzo et al., 2023), the socio-economic dimensions of these measures have received less attention. In a review of high-priority research areas in hydropeaking, Hayes et al. (2023) identified persistent knowledge gaps at the intersection of economics, energy markets, and mitigation practices. Additionally, socio-economic dimensions such as recreational uses, cultural values, and distribution of costs and benefits across stakeholders are rarely incorporated into economic evaluations of hydropeaking mitigation strategies. This omission restricts the development of effective policy frameworks and incentive mechanisms needed for the development of sustainable hydropower. Thus, a comprehensive assessment of the economic aspects of hydropeaking mitigation is essential to balance renewable energy expansion with environmental protection and to support integrated water resource management. This review addresses this gap by synthesizing existing empirical and modeling studies on the economic dimensions of hydropeaking mitigation. Specifically, it aims (i) to evaluate the economic impacts of operational, structural and hybrid mitigation measures, (ii) to assess how effectively these mitigation measures improve ecological conditions, and (iii) to synthesize the methodological approaches used to evaluate the economic and ecological trade-offs in hydropeaking mitigation studies.

Accordingly, we examine the economic dimensions of hydropeaking mitigation with a focus on their policy implications for sustainable hydropower modernization. We first analyze how operational, structural and hybrid mitigation measures affect the economic performance of hydropower operators, which provides insights into how such evidence can inform policy design and market regulation. We then assess the ecological effectiveness of these mitigation approaches, highlighting the types and magnitudes of environmental improvements, including ecosystem services, habitat quality and recreational benefits. Finally, we synthesize the methodological approaches used to evaluate economic and ecological trade-offs in hydropeaking mitigation studies.

## Measures for mitigating hydropeaking

Hydropeaking mitigation encompasses a range of strategies designed to reduce ecological and social impacts of sudden sub-daily flow fluctuations caused by hydropower operations. Such rapid flow changes can degrade aquatic habitat quality, increase fish stranding, and alter ecological processes (Bipa et al., 2024). Mitigation strategies can broadly be classified as operational, constructional and morphological measures (Table 1). Operational strategies such as ramping rate restrictions, restrictions on minimum and maximum flows stabilize flow conditions but can reduce plant flexibility and potential revenue (Guisández et al., 2013; Juutinen et al., 2025). More advanced approaches like the Building Block Approach (BBA) aim to mimic natural flow patterns and enhance aquatic habitat suitability (Kang and Choi, 2019). In some cases, targeted and conditional releases can be more cost-effective than rigid minimum flow rules, as they align better with ecological thresholds (Casas-Mulet et al., 2014).

**Table 1:** Types of hydropeaking mitigation measures

Category	Measures	Key References
Operational	Ramping rate restrictions; minimum environmental flows; targeted/conditional releases; Building Block Approach	Casas-Mulet et al., 2014; Kang and Choi, 2019; Juutinen et al., 2025
Constructional	Re-regulation reservoirs; compensation basins; demodulation galleries; bypass systems; hybrid hydropower–BESS solutions	Tonolla et al., 2017; Chalishazar et al., 2022; Höfkes et al., 2024
Morphological	Side-channel reconnection; floodplain restoration; instream habitat enhancement; channel restructuring	Vanzo et al., 2016; Kopecki and Schneider, 2016; Moreira et al., Premstaller et al., 2017
Policy and Market	Compensation funds; market integration of environmental flow costs; willingness-to-pay; standardized regulatory thresholds for mitigation	Kataria, 2009; Kotchen et al., 2006; Dalcin et al., 2024; Ruokamo et al., 2024

Constructional measures involve physical intervention, such as Re-Regulation Reservoir (RRR), compensation basins, demodulation galleries and bypass systems, which buffer and/or redirect flows to dampen hydropeaking impacts and retain some hydropower flexibility (2019; Bieri et al., 2016; Guisández et al., 2014; Premstaller et al., 2017; Premstaller et al., 2017; Tonolla et al., 2017). Hybrid solutions that combine structural and technological strategies, such as hydropower plants paired with Battery Energy Storage Systems (BESS), are increasingly being recognized as potential alternatives, as they shift flexibility from river flows to energy storage, balancing ecological benefits with system profitability (Chalishazar et al., 2022; Höfkes et al., 2024).

Morphological measures aim to improve riverine resilience to hydropeaking by restoring or modifying channel structures and habitats instead of affecting the flow releases directly. Channel morphology directly influences eco-hydraulic responses to hydropeaking, with braided and transitional reaches resulting in higher habitat diversity and resistance to rapid flow fluctuations compared to single-thread channels (Vanzo et al., 2016). Practical applications of morphological measures include side-channel reconnections, floodplain restoration, and in-stream habitat enhancements that create hydraulic refuges to reduce fish stranding and improve habitat stability (Kopecki and Schneider, 2016; Moreira et al., 2019). For example, a case study from the Valsura River showed that integrating morphological measures with constructive infrastructure can improve ecological outcomes, increase human safety and maintain flexibility of energy production (Premstaller et al., 2017).

Complementary to these technical measures, market-based mechanisms and policy instruments have emerged as approaches to finance and incentivize mitigation (Table 1). Specifically, integrating the costs of environmental flows into electricity markets or creating compensation funds can help distribute the financial responsibility for ecological restoration across society (Dalcin et al., 2024). In addition, evidence on measuring public willingness to pay for environmental improvement can legitimize and strengthen the implementation of mitigation measures even when hydropower profitability is reduced (Kataria, 2009; Ruokamo et al., 2024).

In parallel, regulatory instruments remain the primary means through which hydropeaking mitigation is mandated. These include thresholds embedded in environmental legislation and hydropower licensing conditions. While some European countries, including Switzerland and Austria, have established legal flow-threshold regulations to maintain good ecological status, others regulate hydropeaking on a case-by-case basis (Norway) or include mitigation recommendations in river basin management plans (Spain, Baden-Württemberg-Germany) (Moreira et al., 2019). In North America (Canada and the USA), hydropeaking-specific regulations are absent, and the existing national legal instruments for water management and species protection are applied during power permitting. Recent evidence from Norway indicates that over 3000 km of rivers downstream of 800 hydropower plants lack appropriate mitigation thresholds (Halleraker et al., 2022). Taken together, these approaches highlight a persistent gap between available mitigation knowledge and policy implementation. While regulatory potential exists, hydropeaking governance remains fragmented, which underscores the need for more systematic, comprehensive and standardized policy instruments to guide hydropeaking mitigation globally (Hayes et al., 2023).

## Methodology

### Literature Search Strategy

**Fig. 1:**
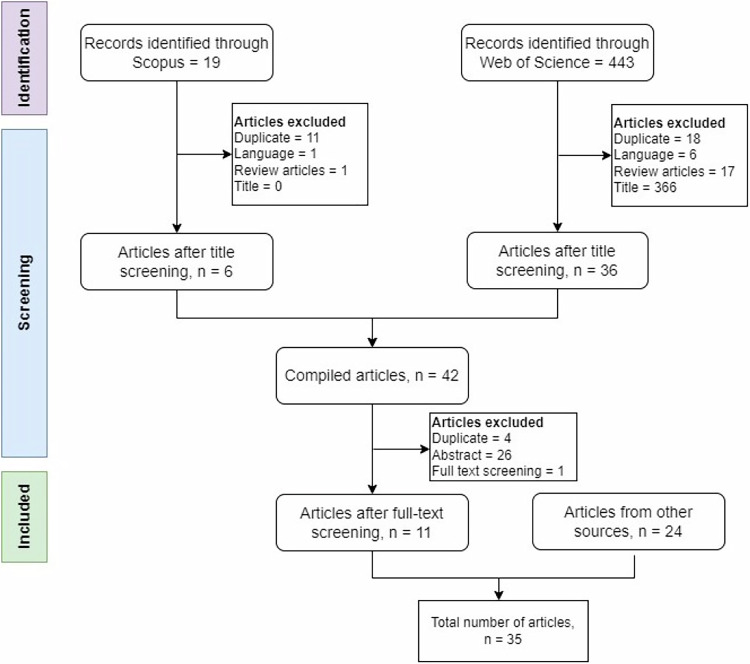
Overview of the PRISMA method for studies related to the economics of hydropeaking mitigation

A comprehensive literature review was conducted following the PRISMA guidelines, a multi-step systematic approach for identifying and selecting relevant research documents. The process involved three main stages: (1) Identification, (2) Screening, and (3) Inclusion (Fig. 1). The literature search was performed on April 14, 2025, using the Scopus database with the search string “TITLE-ABS-KEY” and the Web of Science (WoS) Core Collection using the “Topic” search function. In both databases, keyword searches were conducted using combinations of two different terms connected by “AND” and “OR” operators. The keywords used in the search are shown in the Table 9.

After combining the articles from different search keywords, a total of 19 articles were identified in Scopus and 443 in Web of Science. In addition, several relevant articles were retrieved from Google Scholar using the same set of keywords.

### Screening Process

After removing duplicates, non-English articles, reviews, and titles, 6 articles from Scopus and 36 from Web of Science were selected for abstract and full-text screening. Following duplicate removal, 38 articles were included for abstract screening and then 12 for full-text review, based on their potential focus on the economic aspects of hydropeaking mitigation measures. Ultimately, 11 articles met the criteria and were included in the review. The criteria adopted to select the articles reviewed in this study are shown in Table 10, among those that emerged from the database search. In addition, 24 articles were identified through other sources such as Google Search and Google Scholar.

### Data Extraction and Analysis

The extracted information from the studies was compiled in the synoptic table, where variables such as mitigation type, methodological approach, economic indicators (e.g., cost estimates, revenue changes), and ecological effectiveness were systematically coded. Summary tables and figures were produced by aggregating numerical values, calculating frequency distributions, and grouping studies by country, mitigation category, and methodological approach. A country-wise map was also created in QGIS to illustrate the geographic distribution of the studies. In addition, the current state of knowledge on methodological approaches, analytical tools, modeling frameworks, and the reported economic and ecological outcomes of different mitigation measures was synthesized.

## Results

### Geographic Distribution and Temporal Trends in Hydropeaking Mitigation Research

**Fig. 2:**
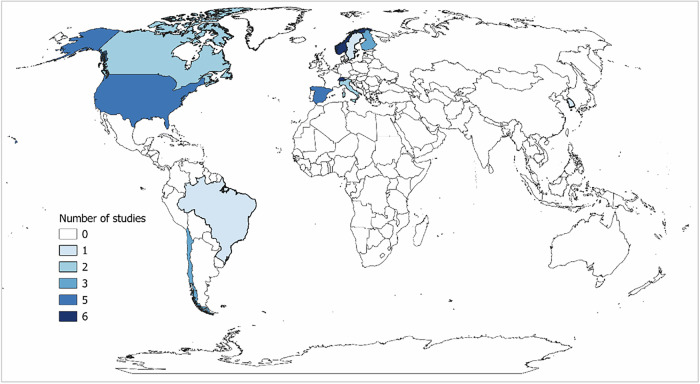
Geographical distribution of studies included in the systematic review. The color gradient indicates the number of studies conducted in each country, with darker shades representing higher representation

The review of the economics of hydropeaking mitigation reveals distinct trends in research focus across time, geography, study type and mitigation measures. Research activity is unevenly distributed across countries (Fig. 2). Within Europe, Switzerland (17%) and Norway (17%) together account for around 34%, reflecting active mitigation research. Other contributions include Spain, about 14%, Finland, about 9%, and Italy, around 6%. Outside Europe, North America, combining the United States and Canada, contributes around 20%, while South America (Chile and Brazil combined) contributes about 11%. However, within Asia, Korea accounts for 3% and no studies were identified from the countries in Africa. This distribution of scientific contributions demonstrates the strong European focus of hydropeaking research, particularly in alpine and temperate river systems.

**Fig. 3:**
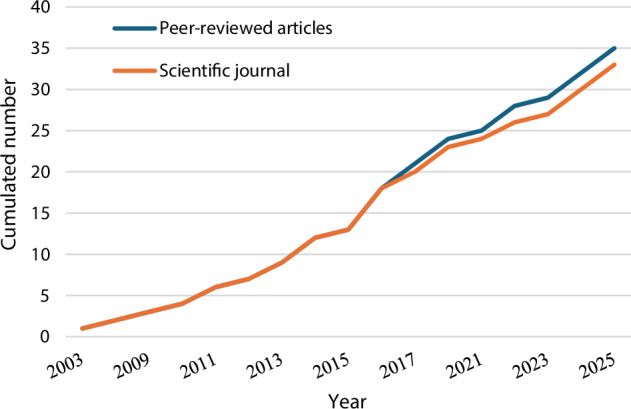
Cumulated number of peer-reviewed articles and the cumulated number of journals publishing these articles

**Fig. 4:**
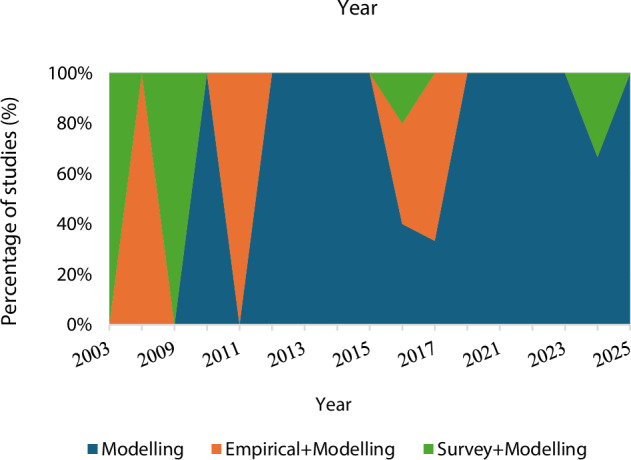
Temporal evolution of study types for hydropeaking mitigation-related articles

The number of studies on hydropeaking mitigation has grown steadily over the last two decades, with an increase after 2010 and a marked surge in 2016, with five studies recorded that year. As shown in Fig. 3, both the cumulative number of publications and the cumulative number of publishing journals continued to grow steadily, indicating a sustained and growing research interest in this topic. The temporal distribution of study types (Fig. 4) reveals that hydropeaking mitigation research has undergone methodological consolidation. Prior to 2013, studies frequently integrated empirical observations or stakeholder surveys with modeling components. Since then, there has been a clear methodological shift toward modeling approaches, which have come to dominate the literature. This trend reflects advancements in computational methods and a growing emphasis on system-level simulation and optimization for hydropower management.

**Fig. 5:**
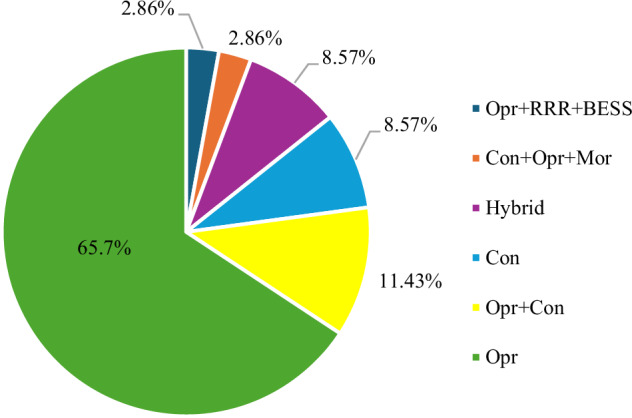
Percentage of studies addressing different mitigation measures for flexible hydropower. Note: Opr Operational, Con Constructional; Mor Morphological, Hybrid: 3 studies considered hybrid differently, BESS with either RRR, HPP or compensation basin

According to the review, operational strategies are the most dominant approach, which account for 23 articles (65.7% of the total), highlighting their practicality and widespread implementation potential in mitigating hydropeaking impacts (Fig. 5). Combined strategies, such as operational and constructional measures were incorporated into 4 studies (11.43%), while constructional measures and hybrid approaches each appear in 3 studies (8.57%). More complex mitigation strategies, such as those combining constructional, operational and morphological measures and those integrating operational, RRR and BESS, are rarely examined, each appearing in only 1 study (2.86%).

The following sections synthesize the findings according to the three research aims. Section 4.2 addresses Aim (i) by presenting the economic impacts of hydropeaking mitigation measures. Section 4.3 addresses Aim (ii) by presenting their ecological effectiveness. Finally, Section 4.4 addresses Aim (iii) by reviewing the methodological approaches used to assess hydropeaking mitigation trade-offs.

### Economic Impacts of Hydropeaking Mitigation Measures

The reviewed literature suggests that while hydropeaking mitigation provides ecological benefits, it is consistently associated with economic trade-offs. Operational, structural and technological measures lead to hydropower revenue loss, increased system costs, or the need for substantial capital investments. The magnitude of these economic impacts varies greatly depending on the type and intensity of the mitigation measures implemented.

#### Economic Impacts of Operational Mitigation Measures

This subsection is structured in two parts. Section 4.2.1.1 synthesizes the direct financial effects of operational mitigation measures on hydropower plant revenues and profits, while Section 4.2.1.2 addresses system-level costs and broader economic externalities associated with these measures. It is important to note that financial effects refer to losses of a specific actor (e.g., operators) while economic effects refer to broader societal gains and losses. Therefore, these measures may cause financial losses for operators while generating overall benefits for society, for example, through improved environmental conditions. The percentages and cost estimates reported in the following subsections come from specific case studies, each with different system boundaries, hydropower contributions, and regulatory contexts. They should not be interpreted as impacts at the national level or for the entire hydropower sector.

##### Financial effects from operational measures

Across studies, operational constraints on hydropower generally reduce revenues by less than 10%, though highly restrictive or combined measures can lead to substantially higher losses (Table 2). The economic magnitude largely depends on how strongly these measures limit operational flexibility. For instance, ramping rate restrictions typically result in modest reductions, often between 0.7 and 5.9% of annual plant revenues (Guisández et al., 2014; Person et al., 2014). Moderate limits, such as 12:1 flow ratios, have minimal impacts (0.7%; €0.8 million), while tighter ratios (e.g., 5:1) can increase losses above 3% (€3.9 million) (Person et al., 2014). Juutinen et al. (2025) reported that tightening ramping constraints from 250 m³/s to 125 m³/s decreased hydropower revenues by 1.12% (€269,000), while public benefits associated with improved environmental flows were estimated at €334,740–€465,260, yielding net gains ranging from €5,740 to €137,260. Similar trends are observed in profit-based assessments, such as ramping rate constraints reduced profits by 0.5–8.3%, with moderate constraints (e.g., 5000-2000 CFS-h) causing about 1–3% daily profit loss, while stricter ramping limits (250 CFS-h) escalate losses to 8% (Niu and Insley, 2016; Niu and Insley, 2013). These effects arise because stricter ramping limits reduce the ability to respond to short-term electricity price spikes, thereby lowering the average selling price of generated power.

**Table 2:** Financial effects from operational measures

Operational measure	Revenue loss	Influencing factors	References
Ramping rate restrictions	0.7–5.9% (€0.8-€2.68 million)	Tighter restrictions cause higher losses, reduced operational flexibility leading to an inability to respond to price spikes, and a consequent decrease in the average electricity selling price.	Guisández et al., 2014; Juutinen et al., 2025
MIF constraints	up to 9% (occasionally up to 50%)	Higher required flows cause greater revenue losses; rigid constant flow leads to higher losses than flexible operations; high price volatility exacerbates the financial impact of these constraints.	Olivares and Lund, 2012; Pérez-Díaz and Wilhelmi, 2010
Combined MIF and MRR	Around 2–11%	The stringency and specific combination of MIF and MRR values; revenue loss is most severe when both constraints are highly restrictive. The impact is minimized when the MRR is less restrictive than the MIF.	Pérez-Díaz and Guisández et al., 2013
Bypass release	0.4–17%	Revenue losses depend on flow volume and release duration. Permanent releases lead to higher loss compared to conditional or seasonal releases	Casas-Mulet et al., 2014
Dam re-operation (naturalized flow)	about 27%	Revenue losses due to shifting toward near-natural flow patterns, which reduces the ability to generate power during high-demand, high-price periods.	Kang and Choi, 2019
Minimum reservoir constraints	0.5-90.3 MNOK/year	Constraint severity, system size, flexibility characteristics, price variability, timing	Mathisen et al., 2022
	**Profit loss**		
Ramping rate restrictions	0.5–8.3%	Profit declines with tighter ramping limits. Stricter ramping constraints shift the down- to up-ramping transition to higher prices.	Niu and Insley, 2016; Niu and Insley, 2013
Min/max discharge constraints	1.1% (from $225,857 to $223,292)	Severity of constraint	Niu and Insley, 2013; Huuki et al., 2022
	**Income loss**		
Minimum reservoir constraints	0.7–133.8 MNOK/year	Constraint severity, system size, price variability	Mathisen et al., 2022

Minimum instream/environmental flow constraints are generally the most influential single factor, with reported revenue losses up to 9% under high price variability and occasionally reaching 50% under highly rigid or constant flow requirements (Olivares and Lund, 2012; Pérez-Díaz and Person et al., 2014; Wilhelmi, 2010; Wilhelmi, 2010). Losses are mitigated when flow requirements are flexible (Huuki et al., 2022) or adaptive to hydrological and market conditions. When maximum ramping rate and minimum flow constraints are combined, annual revenue losses typically reach up to 11%, with particularly severe impacts (>4%) occurring when both constraints are highly restrictive (e.g., >3% Minimum Instream Flow (MIF) and >12 h Maximum Ramping Rate (MRR)) (Guisández et al., 2013). Pérez-Díaz and Wilhelmi (2010) found that when maximum ramping rates are greater than or equal to the minimum flow, the maximum decrease in revenue is only 0.45% compared to scenarios without ramping rate constraints. However, stricter minimum flow requirements lead to smaller revenue losses compared to stringent maximum flow constraints (Huuki et al., 2022).

Other operational adjustments, such as bypass releases and naturalized flow re-operation, show greater variability. Bypass releases cause annual losses between 0.4 and 17% (Casas-Mulet et al., 2014). This wide range reflects several interacting factors: (i) release type - production releases generate revenue that partially offsets costs, whereas pure bypass releases do not, resulting in higher net losses; (ii) timing and duration - permanent or seasonal releases require large water volumes and cause the highest losses (up to 17%), while conditional or targeted releases during critical ecological periods use less water and result in much lower losses (as low as 0.4%); and (iii) opportunity cost - water is valued at peak market prices, thus losses reflect not only volume but also inter-annual and seasonal electricity price variability (Casas-Mulet et al., 2014). Complete dam re-operation to restore natural flow patterns can reduce hydropower benefits by approximately 27% (477,299–517,073 kWh vs. 685,222 kWh under hydropeaking), primarily as such operations eliminate opportunities to generate during high-demand, high-price periods (Kang and Choi, 2019).

##### Economic costs and externalities of operational measures

Beyond these direct impacts, operational mitigation strategies impose significant externalities on the broader energy system and society (Table 3). Ramping constraints can increase operational cost by approximately 20% (Anindito et al., 2019), with a 50% ramping reduction, system balancing costs increased by €59,000, leading to total annual costs of €328,000 (Juutinen et al., 2025). Lower natural gas prices reduce the financial cost of implementing ramping restrictions by nearly 50%, decreasing total restriction costs from $0.5 million to $0.25 million per month (Kern and Characklis, 2017). Environmental flow constraints increased total system costs by less than 2.5% (approximately $30 million/year), with minimum flow restrictions having a greater impact than ramping limits. When combined, these constraints raised system costs by up to 2.32% (Olivares et al., 2015). In a later study, cost increases exceeded 70% under the strictest conditions, while moderate combinations of MIF and MRR constraints led to increases between 10% and 20% (Olivares et al., 2021). Meanwhile, discharge limitations caused the highest production losses, ranging from 2.4–7.2% (€2.9–8.5 million), while increased residual flows of 1–3 m³/s led to annual production losses of 1.4–4.6% (€1.8–5.4 million), making these measures more costly relative to their ecological effectiveness and more expensive than compensation basins (Person et al., 2014). In extreme cases, such as the complete elimination of hydropeaking, the average electricity selling price reduces by 5–10%. Taking this total abandonment as a reference scenario (100% loss), economic losses reach nearly 100% for intermediate basin (150,000 m³) and ~92% for larger basin (400,000 m³). In contrast, reducing hydropeaking to a 1:5 ratio reduces these losses to ~23% and ~10%, respectively (Gostner et al., 2011). Environmental flow implementations led to energy losses up to 18% and the financial burden is also redistributed to consumers, increasing electricity bills by 0.42% for residential users and up to 12% for larger consumers (Dalcin et al., 2024), while substantial compensation funds averaging $129–266 million/year may be required to offset energy losses from sustainable operation (Dalcin et al., 2024). Furthermore, switching from peaking to Run-Of-River (ROR) operation would increase producer costs by approximately $310,612 per year (95% confidence interval mean range: $219k-$402k), due to the need for additional thermal generation. However, the associated social benefits, particularly the estimated use value benefit to recreational anglers, are $738,400 per year ($301,900–$1,068,600), more than double these costs (Kotchen et al., 2006).

**Table 3:** Other evaluated economic aspects due to operational measures

Operational measure	Financial aspect	Range of loss/cost	Influencing factors	References
Ramping constraints	Financial cost	Operational cost increase ~20%, system balancing costs increase by €59,000	Natural gas price fluctuations, Peak/off-peak electricity price spread, stringency of the ramping rate reduction, and CO₂ emissions consideration	Anindito et al., 2019; Juutinen et al., 2025; Kern and Characklis, 2017
MIF and MRR	System/generation cost	Power system cost increases up to 70%	Constraint combination severity; relative impact of MIF (higher) vs. MRR, minimum flow requirement level, system flexibility	Olivares et al., 2015
Combined (MIF+MRR+bypass release)	Electricity price increase	Average 14%	Stringency of constraints (min. flow and bypass: 2–8%; ramping: 15–25%); minimum bypass flows lead to power losses (3%); minimum discharge flow reduces income and flexibility; 4% average hourly ramping reduction	Aubin et al., 2025
Combined (MIF+ minimum reservoir filling)	Production loss	2.5% (3.7 TWh/yr) under stronger reservoir restrictions with standard MIF; Overall national estimate: 3.0-3.1 TWh/yr (∼2%)	Overall estimate includes 285 potential constraints; constraint type (flow vs. reservoir); constraint stringency (standard vs. stronger); flow volume; seasonal timing; reservoir storage capacity; system flexibility; interaction effects	Arvesen et al., 2025
Min/max discharge constraints	Mitigation cost	0.7–2.6% of annual production	Flow constraints level; mitigation implementation cost, annual hydropower revenue and production baseline	Casas-Mulet et al., 2016
Minimum bypass flow	Production loss	3%	Bypass flow volume (2–8% of max flow); stringency of environmental constraints	Aubin et al., 2025
Minimum flow requirement	Production loss	1.8% (2.6 TWh/yr)	Constraint level, flow volume and seasonal timing	Arvesen et al., 2025
Limiting max/drawdown rate, increasing residual flow	Production loss	1.4–7.2% annual production loss (€1.8–8.5 million)	Magnitude of the flow change (m³/s)	Person et al., 2014
Hydropeaking elimination/ratio reduction	Economic loss	5–10% reduction in selling price under total elimination; 10-100% economic loss	Reduction severity, hydropeaking reduction ratio; available basin storage volume	Gostner et al., 2011
Environmental flow implementation	Energy loss and compensation fund	10–18% energy loss; compensation fund: $129–266 M/year (up to $760 M)	Target level of environmental performance; Climate change scenario; Electricity consumption profile and pricing; Scale of required ecosystem restoration	Dalcin et al., 2024
Switching from peaking to ROR	Producer cost	Producer costs increased $310,612/year; recreational fishing benefits: $738,400/year	Cost of replacing lost flexibility with other generation sources (e.g., thermal), recreational and ecosystem benefits	Kotchen et al., 2006
Minimum reservoir constraints	Flexibility factor, power production	0.34–9.6% flexibility loss; 0.6-175 GWh/year production loss	Constraint severity, price variability and system size	Mathisen et al., 2022
Minimum reservoir filling	Production loss	0.4% (0.57 TWh/year)	Constraint level, flow volume and seasonal timing	Arvesen et al., 2025

#### Economic evaluation of structural measures

Constructive or structural measures, including compensation basins, RRRs, retention volumes, and subterranean channels, are designed to mitigate hydropeaking impacts through physical infrastructure. Costs vary with design and capacity (Table 4), ranging from €0.6–4.5 million/year for 50,000–1,000,000 m³ compensation basins (Olivares et al., 2021; Person et al., 2014) to over CHF 25 million and over CHF 15 million for 30,000 m³ caverns and basins, respectively (Bieri et al., 2016). RRR represents a cost-effective and profitable solution at $2–25/m³, with total investments reaching up to $9 million for a 0.36 million m³ capacity reservoir, and their economic attractiveness is underscored by Internal Rates of Return (IRR) often exceeding 25% (Anindito et al., 2019). According to Premstaller et al. (2017), a demodulation gallery combined with a new penstock increased production capacity to 8 MW and annual generation to 9.2 GWh. The measure also increased peak energy flow from 60% to 88% and minimize residual hydropeaking from 40% to 2%, but at a high investment cost (Premstaller et al., 2017). The use of subterranean channels can also substantially reduce economic losses from 100% under complete peak elimination to 3–44%, depending on channel length. For example, a 0.5 km underground channel results in only 3% economic loss, while extensions up to 12.5 km channel can incur losses up to 44% (Gostner et al., 2011).

**Table 4:** Economic aspects of structural mitigation measures

Constructive measure	Range of cost / economic effectiveness	Influencing factors	Reference
Compensation basins	€0.6–4.5 million/year for 50k–1000k m³ basin; >CHF 15 million for 30k m³ basin; >CHF 25 million for 30k m³ cavern	Basin volume, design type (open vs. cavern), cost–benefit optimization	Bieri et al., 2016
Multipurpose compensation basin (linked to new HPP)	40 GWh/year (10 MW) production; cost-effective while maintaining economic sustainability	Integration with ROR generation	Meile et al., 2011
Retention volume	80,000 m³ volume is more cost-effective than a 100,000 m³	Storage capacity, trade-off between cost and ecological efficiency	Tonolla et al., 2017
New HPP + demodulation gallery + penstock	Enables 8 MW power and 9.2 GWh/year generation. Increases peak energy flow from 60% to 88%, reduces residual hydropeaking from 40% to 2%	Construction complexity, operational flexibility and residual hydropeaking volume	Premstaller et al., 2017
Subterranean channel	Economic loss reduced from 100% (total hydropeaking elimination) to 3–44%	Channel length, flow diversion capacity	Gostner et al., 2011
RRR	$2–25/m³ (total $0.72–9 million for 0.36 Mm³); highly cost-effective and profitable	Reservoir capacity, unit construction cost	Anindito et al., 2019
RRR + MIF + MRR (combined solution)	Small RRR (0.5-2h capacity) limit system cost increases to below 5-10%; without RRR, cost increase 70% (strict) and 10–20% (moderate)	Reservoir capacity, constraint level (MIF/MRR), system flexibility	Olivares et al., 2021

#### Economic Evaluation of Technological Measures

Table 5 highlights that recent technological innovations offer complementary solutions to structural and operational adaptations. BESS are emerging as cost-effective tools where land availability, construction costs, or social constraints limit reservoir feasibility, and can achieve IRRs >10–15% beyond 2025, especially under stringent ramping restrictions (Anindito et al., 2019; Person et al., 2014). Hybrid systems combining 10–100 MWh BESS with basins reduce required storage volume by 51–96%, with equivalent replacement costs of CHF 350–5600/m³ and BESS investment and maintenance costs of 5577–21,784 kCHF and 84–263 kCHF, respectively (Höfkes et al., 2024). Integration of BESS with hydropower operations also generates additional revenues up to $1.2 million. Peaking operations with high-power and short-duration BESS (60MW/2h) yield the highest net revenue ($26.6 M vs. $25.4 M without BESS) (Chalishazar et al., 2022). Finally, an advanced optimization approach that incorporates both ramping constraints and Transition Cost cuts (TC-cuts) leads to a reduction in profits. In the unconstrained case, profit was €7,221 million, which decreased slightly to €7,220 million when only ramping constraints were introduced. Incorporating ramping constraints together with the strictest TC‑cuts further reduced profit to €7,159 million (Helseth et al., 2023).

**Table 5:** Economic aspects of technological measures

Technological measure	Economic aspects	Influencing factors	Reference
Fixed technical cost	Cost of starting a hydropower plant: 200 €	Frequency of plant start-ups, operational schedule	Casas-Mulet et al., 2014
BESS	Cost-effective from ~2025 onward (IRR >10–15%); profitable mainly under stringent MRR conditions; lower-cost alternative to environmental flows; competitive when RRRs are infeasible or costly	Electricity market conditions, ramping constraints (MRR), RRR feasibility, land/social constraints	Anindito et al., 2019
Hybrid (BESS + basin)	Initial BESS investment: 5577–21,784 kCHF; maintenance cost: 84–263 kCHF; extra production cost: 0.5–7.3 kCHF; equivalent prices: 350–1000 CHF/m³ (Francis), 1100–3000 CHF/m³ (Pelton); reduces basin volume 51–96%; cost-competitive under high basin investment or limited space	BESS size, turbine type, basin construction cost, lifetime, discount rate, stringency of environmental requirements	Höfkes et al., 2024
Hybrid (Operations + BESS)	Generate additional revenue up to $1.2 M	BESS configuration (power × duration), flow pattern (peaking, intermediate, run-of-river), electricity market prices	Chalishazar et al., 2022
Ramping constraints with TC-cuts	Slight profit decrease: from €7221 M (baseline) to €7220 M (ramping only) and €7159 M (Ramp-TC100); computation time increases substantially (over 3x)	Ramp constraint severity, inclusion of transition cost cuts, computational complexity	Helseth et al., 2023

#### Public Willingness to Pay for Hydropeaking Impact Mitigation

Willingness to pay for reducing hydropeaking impacts and improving river ecosystems has been documented across several countries. In Finland, households are willing to pay €29–54 annually to reduce hydropeaking impacts, particularly for improved fish stocks, recreational opportunities, and ecological health (Ruokamo et al., 2024). The average Swedish household is willing to pay 1100-1400 SEK/year for the improvement of single environmental attributes (fish stock, bird life, benthic invertebrate richness, and river-margin vegetation and erosion) and approximately 2100 SEK/year for the improvement of a combination of these attributes (Kataria, 2009). Similarly, in the U.S., nearly half of surveyed participants supported an annual payment of $25 for ecological improvement from operational changes in hydropower systems (Jones et al., 2016). On a larger scale from Finland, Juutinen et al. (2025) found that the aggregate annual Willingness To Pay (WTP) for ecosystem improvements under moderate Environmental Flow (EF) constraints ranged from €334,740 with increased CO₂ emissions to €465,260 without CO₂ emissions, highlighting greater public support when EF policies align with low-emission energy solutions.

#### Recreational Economic Benefits of Flow Enhancement

Enhancing the river flow in the Ticino River was estimated to increase the seasonal consumer surplus for recreational anglers from 925 CHF to 1364 CHF per individual, representing a gain of approximately 440 CHF. When extrapolated to the estimated population of 3000 anglers, the total annual economic benefit amounts to approximately 1.32 million CHF (Buchli et al., 2003).

#### Economic Implications Across Power Plant Capacities

**Fig. 6:**
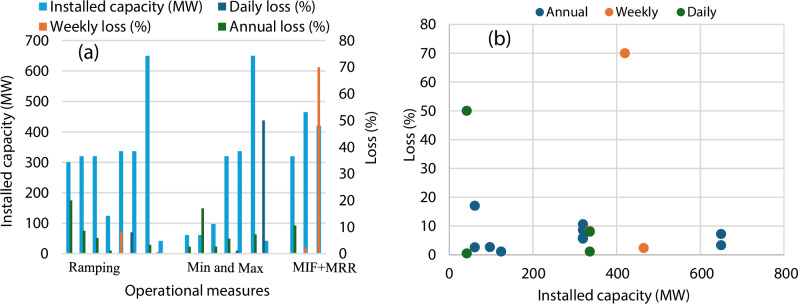
Panel **a**) represents the economic losses (%) for different operational measures across plant sizes. Panel **b**) represents the economic losses (%) at different time scales across plant sizes.

This section synthesizes the evidence of how power plant capacity influences the magnitude and distribution of economic impacts associated with hydropeaking mitigation measures. The evaluation of operational measures shows that profit losses vary depending on the temporal scale and plant size (Fig. 6a). Plants with higher installed capacities tend to experience lower relative annual losses, suggesting better efficiency and economic resilience at scale. In contrast, smaller or more constrained plants show notably higher weekly and daily losses, indicating greater sensitivity to operational limitations. Across the evaluated strategies, profit reductions remain within a moderate range, with maximum losses of approximately 10% (Fig. 6b). While daily and weekly operational losses exhibit high variability, the annual profit loss converges to a more moderate and consistent rate of approximately 10%, largely independent of the plant's installed capacity. The combined MIF and MRR measures exhibit the greatest impact on generation efficiency, highlighting the need for optimized operational flexibility to maintain profitability across varying time scales.

The results summarized in Table 6 demonstrate variation in construction costs and economic impacts across hydropower plant sizes. Smaller plants, such as the 4.7 MW installation, require higher investment relative to their capacity, with construction costs exceeding CHF 15–25 million for a 30,000 m³ compensation basin. Moderate-capacity plants (around 300 MW) show construction costs ranging from $0.7 - $9 million for a 0.36 million m³ reservoir, whereas larger plants (650 MW) exhibit annual costs of €0.6–4.5 million for compensation basins of 50,000–1,000,000 m³. Although the reported values vary in units, the findings suggest that smaller plants may face proportionally higher cost burdens relative to their installed capacity. However, due to the heterogeneity of cost units and reporting approaches across studies, this comparison should be interpreted qualitatively rather than as a statistically established relationship. This finding, however, contrasts with the analysis by (Venus et al., 2020), who reported a positive correlation between hydropower plant capacity and the absolute construction costs of upstream fish passage measures in Europe. The apparent discrepancy likely arises because our review synthesizes heterogeneous, case-specific cost information across plant sizes without formal normalization, while Venus et al. (2020) focused on absolute costs for site-specific fish pass installations.

**Table 6:** Costs and economic impacts of constructional measures across hydropower plant sizes

Plant size (MW)	Construction cost
4.7	CHF 15–25 million (30,000 m³ basin)
183	3-44% economic loss (subterranean channel)
300	$0.7–9 million (0.36 million m³ RRR)
420	<8% cost increase (30-minute RRR)
650	€0.6–4.5 million/year (50k–1,000k m³ basin)

### Ecological Effectiveness of Hydropeaking Mitigation Measures

The findings summarized in Table 7 highlight that different hydropeaking mitigation strategies provide varying degrees of ecological benefits. Operational measures, such as ramping rate restrictions, have been demonstrated to provide multiple ecological benefits, such as protecting aquatic ecosystems and generating environmental gains (Niu and Insley, 2016; Niu and Insley, 2013), reducing flow fluctuations (Olivares et al., 2021) and fish stranding risk (Premstaller et al., 2017), moving regulated flows closer to natural regimes and lowering flashiness, thereby improving ecological conditions (Anindito et al., 2019). Flow constraints, such as minimum flow releases and reduced discharge during spawning, are particularly effective for egg and alevin survival and reduce early life stage mortality of salmonids, with earlier and longer reductions yielding higher survival rates (Casas-Mulet et al., 2016). However, while low flows can enhance habitat suitability (Person et al., 2014), imposing significantly low constant flows, such as fixed annual minimum discharges (e.g., flows exceeded on approximately 347 days/year in the historical record) may cause environmental harm by reducing wetted area, limiting habitat availability and suppressing natural intra-annual variability that provides critical ecological cues for aquatic and riparian species (Bejarano et al., 2019). The transition from hydropeaking to ROR operation resulted in significant ecological benefits, including increased Chinook salmon habitat and population growth (Kotchen et al., 2006). Similarly, dam re-operation to restore a more natural flow regime created similar aquatic effects, such as 18.6% increase in Weighted Usable Area (WUA), about 5% increase in aquatic habitat, and consistently higher habitat suitability indices under the restored flow condition compared to hydropeaking (Kang and Choi, 2019).

**Table 7:** Ecological effectiveness of hydropeaking mitigation measures

Mitigation measure	Ecological effectiveness	Reference
Ramping restrictions	Protect aquatic ecosystems; reduce stranding risks, improve environmental flows; reduce fluctuations; lower flashiness; achieve flows closer to natural regimes; area affected by hydropeaking reduced by 3%; minor improvements in hydraulic safety for recreation	Niu and Insley, 2016; Niu and Insley, 2013; Kern and Anindito et al., 2019; Juutinen et al., 2025
Flow constraints/ min/max flow restrictions	Reduce early life stage mortality; enhance egg and alevin survival; benefit the ecosystem; reduce stranding and drift risk, increase fish population and macrozoobenthos density, improve fish reproduction; increase habitat suitability, and fish habitat recovery	Person et al., 2014
Minimum reservoir filling	Enhances recreational opportunities, preserves landscape aesthetics during summer, and maintains littoral zone productivity	Arvesen et al., 2025
MIF + MRR	Reduce flashiness by up to 90%; align flow with natural regime; stabilize flows; improve ecological performance	Olivares et al., 2015
Combined constraints (MIF+MRR+bypass release)	Alleviate riverbed drying; reduce impacts from hydropeaking operations; revenues of unconstrained plants increased by up to 10% due to higher market prices	Aubin et al., 2025
Switching from peaking to ROR	Improves salmon habitat and increases population; provides economic benefits to anglers ($738,400 per year)	Kotchen et al., 2006
Dam re-operation	WUA increased by about 18.6%; aquatic habitat by about 5%, increase Composite Suitability Index	Kang and Choi, 2019
Basins/compensation basins	Restore natural flow regime; reduce daily fluctuations; support flood control and recreation; best ecological rating at braided reach, and improve transient flow conditions	Bieri et al., 2016
Retention volume (basin + cavern)	Reduces ramping rates; lowers stranding and drift; improves fish productivity; improves fish refuge response, stabilizes temperature; higher ecological benefits with larger volume	Tonolla et al., 2017
RRR	Reduces flashiness; smooths downstream releases	Olivares et al., 2021
Demodulation gallery + bypass HPP	Improves ecological and safety conditions; flexible energy production, reduces hydropeaking and discharge variation	Premstaller et al., 2017
Ramping constraints with TC-cuts	Reduces fluctuation and discharge ramping frequency; smooths operation	Helseth et al., 2023
Morphology enhancement	Improves habitat condition/stability; increases suitability in braided reaches; benefits biodiversity; greater improvements with higher residual flow and basin size	Gostner et al., Person et al., 2014
Hybrid (operations + BESS)	Enables environmentally friendly operation; reduces ramping; mitigates hydropeaking impacts	Chalishazar et al., 2022
RRR or BESS	Provides smoother flow operation	Anindito et al., 2019

Structural measures such as compensation basins, RRRs and retention volumes also demonstrate strong ecological performance. RRRs reduce flashiness by smoothing flow releases (2011; Bejarano et al., 2019; Casas-Mulet et al., 2016; Characklis, 2017; Meile et al., 2011; Olivares et al., 2021; Olivares et al., 2021; Olivares et al., 2021; Person et al., 2014; Premstaller et al., 2017; Premstaller et al., 2017), while retention volumes, particularly larger one decreases flow ramping rates, leading to improved refuge conditions during hydropeaking. For example, Tonolla et al. (2017) found that retention volumes of 80,000 m³ greatly reduced flow ramping rates, which reduced risks of stranding for juvenile trout and drift for macroinvertebrates, leading to improved fish biomass and overall fish productivity. Compensation basins are also effective in restoring ecologically flexible flow regimes. For example, multipurpose basins not only reduce daily flow fluctuations to below 0.5 on 84% of days, compared to 53% without a basin, but also attenuate flood flows, support biotope creation and recreation opportunities, while still enabling electricity generation of 40 GWh annually with continued economic viability (Meile et al., 2011).

Compensation measures at the reach scale confirmed several benefits. Person et al. (2014) stated that in braided river reaches, compensation basins yielded the highest ecological ratings, while measures such as limiting drawdown ranges combined with increasing residual flows contributed to maximal ecological benefits, though ecological benefits were often proportional to costs. While in channel reach (a monotonous and straight river section) under low flow and discharge limitation conditions, compensation basins lead to a higher Suitability Habitat Ratio (SHR), defined as the ratio of suitable area to effective wetted area (Person et al., 2014). Hybrid solutions, combining hydropower with BESS, enabled smoother plant operation and reduced ramping mileage, offering a potential option to minimize ecological impacts while maintaining system flexibility (Chalishazar et al., 2022; Anindito et al., 2019).

### Methodological Approaches used to Assess Hydropeaking Mitigation Trade-offs

The study of hydropeaking mitigation integrates a variety of modeling frameworks, field investigations and optimization techniques. This section synthesizes the methodological approaches used to evaluate ecological, hydrological and economic trade-offs, summarized in Table 8 (the extended table of methods and platforms based on different study types is provided in Table 11). Hydrothermal dispatch and hydro-economic models are frequently formulated as Mixed-Integer Linear Programming (MILP) or coupled with Stochastic Dual Dynamic Programming (SDDP) to simulate hydropower system operations under environmental constraints (Anindito et al., 2019; Olivares et al., 2021).

**Table 8:** Summary of methods, tools and platforms used for economic assessment of hydropeaking mitigation measures (extended version is in Table 11)

**Statistical Method/analysis**	Regression models; non-parametric pairwise comparisons; Kruskal–Wallis test (H statistic); Principal Component Analysis; Wang transform; Burn analysis; Monte Carlo simulation; Diagnostic analysis; Mann–Kendall and Spearman’s Rho tests; Discrete choice regression models; Probit model; Random parameter logit; Random Utility Model
**Ecohydraulic model**	River2D model, CASiMiR habitat model, HYDRO_AS-2D, HEC-RAS, HEM-PEAK
**Optimization/dispatch models**	MILP; Stochastic models; Incremental dynamic programming; Discrete dynamic programming; Backward-recursive equation; Hamilton–Jacobi–Bellman partial differential equation; Hydrothermal dispatch models; Grid-wide power dispatch models; ANNs; MOEA; Regime-switching models; Deterministic short-term hydrothermal dispatch coordination model; Power–discharge piecewise linear curves; Optimization models
**Economic model**	WTP, Discrete Choice Experiment, Random utility model, Nested logit model, Hypothetical Travel Cost Model, Contingent Valuation survey approach
**Modeling platforms/Software tools**	nMAG hydropower simulation programme, MATLAB, ProdRisk software, STATGRAPHICS, HEC-RAS, ESRI ArcMap, BASEMENT, CASiMiR, R-programming, LIMDEP econometric software, Nlogit

Ecological impacts are commonly assessed using hydraulic modeling platforms, such as HEC-RAS, River2D, HYDRO_AS-2D and BASEMENT, which simulate flow depth and velocity under different operating scenarios and are combined with habitat models like CASiMiR to evaluate species-specific suitability indices and stranding risks (Casas-Mulet et al., 2014; Kang and Choi, 2019; Person et al., 2014; Premstaller et al., 2017; Tonolla et al., 2017).

Hydrologic alterations are typically quantified through statistical descriptors such as the Richards-Baker Flashiness Index, hydropeaking indicators, ramping ratios and flow flashiness indices (Olivares et al., 2015; Olivares et al., 2021). Advanced optimization and simulation studies have incorporated advanced approaches using MATLAB, GAMS/CPLEX and STATGRAPHICS for statistical processing, while geospatial analyses using ArcMap (Guisández et al., 2016; Juutinen et al., 2025; Guisández et al., 2014). Additionally, numerical approaches have been developed to measure ecological-flow relationships and to optimize mitigation trade-offs under uncertainty conditions employing Artificial Neural Networks (ANNs) and Multi-Objective Evolutionary Algorithms (MOEAs) (Dalcin et al., 2024).

## Discussion

### Key Socio-Economic and Ecological Trade-offs For Hydropeaking Mitigation

This systematic review identifies complex socio-economic and ecological trade-offs inherent in hydropeaking mitigation. The analysis of spatiotemporal trends shows that research activity has grown substantially since 2010, with a strong concentration in Europe and reliance on modeling studies. This regional focus reflects Europe’s advanced hydropower sector and stringent ecological regulations, but also underscores significant geographical gaps in other regions where hydropower is rapidly expanding. The economic assessment shows a clear trade-off between ecological protection and financial performance. Operational measures such as ramping restrictions and minimum flow requirements are easy to implement but lead to direct revenue losses by limiting market flexibility. In contrast, structural and hybrid measures, including RRRs, compensation basins, and BESS, require substantial upfront investment and rely on emerging technologies. However, they offer better long-term ecological and economic feasibility and represent a promising way to decouple energy system flexibility from its impacts on river systems. Importantly, these losses largely represent financial impacts on hydropower operators rather than net economic losses for society. Several studies show that while mitigation measures may reduce hydropower revenues, the associated ecological improvements generate broader societal benefits, including improved ecosystem services, recreational opportunities, and enhanced river ecosystem quality. When these benefits are considered, hydropeaking mitigation may result in positive net welfare outcomes, even if the financial incentives for operators alone appear negative.

Furthermore, the societal WTP underscores significant public value for improved river ecosystems, providing a compelling economic rationale for policy frameworks that incentivize mitigation and redistribute costs. Ecological assessments further confirm that all mitigation approaches significantly enhance habitat stability, reduce fish stranding, and improve flow naturalization. These findings highlight that hydropeaking mitigation is characterized by measurable economic-ecological trade-offs that can be optimized through integrated planning, innovative market mechanisms, and supportive policy frameworks.

### Policy and Governance Implications of Hydropeaking Mitigation

The economic trade-offs identified in this review are not merely operational challenges; they represent a fundamental policy trade-off at the core of the renewable energy transition. Balancing the flexibility needs of a decarbonizing grid with the ecological integrity of river ecosystems requires a sophisticated integration of economic incentives, regulatory frameworks, and collaborative governance.

#### Market Design and Internalizing Externalities

The observed profit losses of 0.5–8% for operational measures (Niu and Insley, 2016; Niu and Insley, 2013) illustrate the need for market mechanisms that internalize environmental externalities. Dalcin et al. (2024) propose an electricity market-based compensation fund where energy consumers contribute to ecosystem restoration, with costs ranging from cents for residential users to thousands of dollars for industrial facilities. Juutinen et al. (2025) further support this approach by demonstrating that the costs of environmental flow policies can be comparable to consumers' WTP for ecological improvements, especially if system balancing can be achieved without increased CO_2_ emissions. This aligns with EU’s Renewable Energy Directive (EU) 2018/2001 (European Union, 2018), which encourages flexible yet sustainable renewable deployment. These studies collectively highlight the urgent need for sophisticated market mechanisms that transparently integrate ecological considerations into hydropower economics.

#### Licensing, Permitting, and Concession Renewals

Studies show that hydropeaking mitigation can be both cost-effective and ecologically beneficial. Casas-Mulet et al. (2016) demonstrate that the implementation costs of operational mitigation measures are relatively low compared to annual production revenue (0.7% to 2.6%), and these measures can be effective in reducing early life stage mortality, suggesting targeted environmental flow releases can inform licensing and permitting by integrating ecological flow requirements into concession renewals.

Barillier et al. (2021) similarly argue for a *“consensual technico-economic framework”* that balances ecological preservation with hydropower flexibility and accounts for complex socio-ecosystem interactions during licensing processes. The ecological effectiveness and long-term economic feasibility of structural measures like re-regulation reservoirs and compensation basins (Olivares et al., 2021; Person et al., 2014) imply that hydropower licensing and concession renewals should explicitly integrate ecological flow requirements and incentivize infrastructure adaptation. The EU Water Framework Directive (2000/60/EC) (European Union, 2000), which aims to achieve good ecological status in water bodies, provides the legal foundation for hydropeaking mitigation even if it doesn’t mention the hydropeaking problem explicitly. Some national permitting frameworks, such as Austria and Switzerland, have already operationalized this goal by incorporating hydropeaking flow thresholds (Moreira et al., 2019). To ensure consistent implementation across Member States, other national permitting authorities should utilize the river basin management framework to mandate site-specific hydropeaking mitigation measures as conditions for new hydropower licenses or the relicensing of existing facilities (Moreira et al., 2019; Halleraker et al., 2022). Our finding that hybrid hydro-BESS systems can balance ecology and profitability (Chalishazar et al., 2022; Anindito et al., 2019) further suggests that permitting processes should be optimized to encourage such innovative, multi-benefit investments, aligning system reliability and flexibility with environmental goals.

#### Incentive Schemes and Funding Mechanisms

Despite the long-term viability of structural and hybrid measures, they require significant capital investment which poses a barrier to implementation. The evidence on societal WTP and the potential for compensation funds for ecological improvements (Dalcin et al., 2024; Juutinen et al., 2025; Kataria, 2009) highlights the need for innovative financing mechanisms that distribute costs equitably among beneficiaries. Payment for Ecosystem Services (PES) schemes could be established, where downstream water users, recreational industries, or even a small surcharge on electricity bills collectively fund mitigation measures, which recognize the public benefits of healthy rivers. Introducing environmental cost-reflective pricing or green certificates could further offset flexibility losses for operators while promoting ecological compliance (Ruokamo et al., 2024; Juutinen et al., 2025). This aligns with the visions of the EU Biodiversity Strategy for 2030 (European Union, 2020), which promotes investing in nature and integrating its value into public and private decision-making. Such funds can reduce the risk of investments for operators and accelerate the adoption of mitigation technologies.

#### Cross-Sector Governance and Coordination

Finally, the spatial and technical complexity of hydropeaking mitigation reveals that technical and economic solutions are insufficient without effective governance. The persistent disconnect between scientific understanding of hydropeaking impacts and policy implementation, as highlighted by Hayes et al. (2023), reflects insufficient cross-sectoral collaboration and policy integration. Effective hydropeaking mitigation thus requires cross-sectoral coordination between energy regulators, river basin authorities, and environmental agencies. Effective planning requires collaboration between energy planners and water managers to assess the cumulative impacts of multiple plants at the river basin scale, rather than the case-by-case approach that is still prevalent in many countries. Integrating hydropeaking mitigation within broader policy frameworks, such as the EU Climate Adaptation Strategy (European Union, 2021), which addresses climate resilience in water management, can foster this necessary collaboration. By creating formal platforms for dialogue and joint planning, governance structures can ensure that hydropower development and modernization advance energy transition goals while fulfilling the legal and ethical commitment to river sustainability.

## Conclusion

This review demonstrates that hydropeaking mitigation is characterized by complex trade-offs between hydropower profitability and ecological effectiveness. Operational measures such as ramping restrictions and minimum discharge are relatively easy to implement and provide significant ecological benefits, although they may constrain revenue opportunities by restricting flexibility in operations in volatile electricity markets. On the other hand, structural measures such as RRRs and compensation basins commonly provide better ecological benefits while still maintaining the opportunity for market activity, but require substantial capital investment and face site-specific feasibility constraints. Hybrid approaches, particularly the integration of BESS with hydropower, prove to be potential options to achieve a trade-off between environmental targets and system flexibility.

Despite recent advances, current analyses of hydropeaking mitigation remain constrained by site-specific case studies, short-term indicators, and the limited incorporation of non-market values and long-term ecological processes. Consequently, evaluations tend to quantify immediate costs and revenue impacts, whereas broader and longer-term ecological and societal benefits are only partially represented, leading to mitigation costs being often overstated and benefits undervalued.

The review further indicates limited integration between hydro-economic models and representations of ecohydraulic dynamics, geomorphic processes, and climate-related uncertainty. Consequently, trade-offs between short-term operational flexibility, long-term ecological dynamics and economic performance remain only partially quantified. Strengthening the linkage between hydro-economic models and ecological response indicators, particularly across temporal scales and under uncertainty, would enhance the robustness and transparency of trade-off analysis.

The geographical distribution of studies is uneven, with most evidence derived from Europe and North America. Expanding empirical analysis to rapidly developing hydropower regions such as Asia, Africa, and South America, where investment is increasing but decisions on mitigation strategies remain underexplored, could improve the generalizability of findings and provide comparative insights into institutional and market differences. Moreover, a critical gap lies in monetizing ecological and social benefits, including biodiversity gains, cultural services, and recreational values, to achieve more balanced and policy-relevant cost-benefit assessments.

Hybrid solutions, such as hydropower–battery storage systems, appear in several studies as emerging options; however, their empirical evidence from real-world demonstration projects to validate economic feasibility, ecological performance, and scalability under different market and policy contexts remains underexplored. Enhanced stakeholder participation and attention to the distribution of costs and benefits across user groups will further improve the social acceptability and policy relevance of economic evaluations. Overall, the review highlights that hydropeaking mitigation is not just a technical operational adjustment, but a multidimensional challenge involving economic and ecological considerations, shaped by market structures, regulatory environments, and site-specific river characteristics. By addressing these gaps, hydropeaking mitigation can evolve from a narrow technical trade-off into a holistic strategy that aligns renewable energy production, ecological integrity, and societal well-being.

## Data Availability

No datasets were generated or analysed during the current study.

## Appendix

**Appendix 1:** Searching keywords

Keywords	Scopus	WOS
Economic* AND benefit* AND hydropeak* AND mitigation*	6	3
Cost* AND benefit* AND hydropeak* AND mitigation*	5	4
Economic* AND benefit* AND hydropeak* AND improve* AND flow*	3	2
Cost* AND benefit* AND hydropeak* AND improve* AND flow*	2	2
Economic* AND benefit* AND hydropeak* AND ramp* AND restriction*	0	1
Cost* AND benefit* AND hydropeak* AND ramp* AND restriction*	0	1
Economic* AND benefit* AND hydropeak* AND flow* AND restriction*	1	2
Cost* AND benefit* AND hydropeak* AND flow* AND restriction*	1	2
Economic* AND benefit* AND hydropeak* AND flow* or ramp* AND restriction*	1	426

**Appendix 2:** Inclusion criteria for reviewing the studies

Criteria	Inclusion	Exclusion
Context	Only records containing all selected keywords were retained	Records containing only one keyword or unrelated to the search were discarded
Language	English	Other languages
Types of study	Peer-reviewed articles, conference proceedings	Review articles, editorial materials, book chapters
Study focus	Economic aspects of hydropeaking mitigation measures	Impacts of hydropeaking, hydropeaking characterisation
Access	Records with a specific direct link for access were considered	Records with broken and no direct link were excluded

**Appendix 3:** Summary of methods, tools and platforms used for economic assessment of hydropeaking mitigation measures

Study type	Statistical Method/analysis	Ecohydraulic model	Optimization / dispatch models (decision-making and planning tools)	Economic model	Platform/tools	Author
**Modeling**	Linear regressions, Stepwise linear regression, non-parametric pairwise comparisons, Kruskal-Wallis tests with the H statistic, Principal Component Analyses (PCA), Cubic smoothing splines, Regression models, First-order assessment method, Regression models, Multiplicative aggregation method, Wang transform, Burn analysis, Monte Carlo simulation, Richard-Baker flashiness index, Diagnostic analysis	River2D model, Habitat Suitability Index (HSI) model, CASiMiR habitat model, HYDRO_AS-2D, HEC-RAS, HEM-PEAK, Hydrologic model, Hydrodynamic model	MILP/MIP, Linear programming (LP), Stochastic model, Stochastic linear programming, SDDP, Incremental dynamic programming (IDP), Discrete dynamic programming (DDP) / Incremental DDP, Backwards-recursive equation, Hamilton Jacobi Bellman-Partial Differential Equation, Deterministic/stochastic dynamic optimization models, Hydrothermal dispatch models, Grid-wide power dispatch models, ANNs, MOEA, Dynamically Adaptive Environmental Flows (DAE-flows), TC-cuts, CPLEX solver, Master-slave algorithms (IDP + MILP), Karush–Kuhn–Tucker conditions, Electricity price models, Regime-switching models, Climate models (Eta-MIROC5, Eta-HADGEM2-ES), Deterministic short-term hydrothermal dispatch coordination model, Power-discharge piecewise linear curves (PDC), Optimisation models.	WTP, Choice experiment (CE)	nMAG hydropower simulation programme, MATLAB, GAMS, AMPL, CPLEX, ProdRisk software, STATGRAPHICS, HEC-RAS 6.3 software, ESRI ArcMap software, CONOPT with GAMS	Anindito et al., 2019; Bejarano et al., 2019; Casas-Mulet et al., 2014; Chalishazar et al., 2022; Dalcin et al., 2024; Guisández et al., 2016; Guisández et al., 2014; Guisández et al., 2013; Juutinen et al., 2025; Helseth et al., 2023; Höfkes et al., 2024; Huuki et al., 2022; Kang and Choi, 2019; Kern and Characklis, 2017; Niu and Insley, 2016; Niu and Insley, 2013; Olivares et al., 2021; Olivares et al., 2015; Olivares and Lund, 2012; Pérez-Díaz and Wilhelmi, 2010; Person et al., 2014
**Empirical + Modeling**	Linear regression model, Regression analysis, Pardé coefficients, Mann–Kendall and Spearman’s Rho tests	Crisp model, CASiMiR model, Hydraulic model, 2D HYDRO_AS-2D, Hydrological runoff model, Fishery population model, Air2stream model		Cost-benefit analysis, Ex Post Benefit-Cost Analysis, Gomez and Probst method, Random utility model, Michigan Recreational Angling Demand Model, Nested logit model	nMAG hydropower simulation program, BASEMENT software, CASiMiR software, Hydropark software, R-programming	Casas-Mulet et al., 2016; Gostner et al., 2011; Kotchen et al., 2006; Meile et al., 2011; Premstaller et al., 2017; Tonolla et al., 2017
**Survey + Modeling**	Regression, Discrete choice regression models, Probit model, Conditional logit (CL) model, Random parameter logit (RPL), Mixed logit (MXL), Random Utility Model (RUM), Lognormal distributions, Correlated utility coefficients, Log-sum formula, Krinsky–Robb method, Bayesian D-efficient design, Decision tree framework			Hypothetical Travel Cost Model (HTCM), Contingent Valuation (CV) survey approach, Discrete Choice Experiment (DCE), Choice experiment, WTP	LIMDEP econometric software package, MATLAB, Nlogit 3.0 software	Buchli et al., 2003; Jones et al., 2016; Kataria, 2009; Ruokamo et al., 2024

**Appendix 4:** Characteristics of the studied power plant

Author	HPP name	Installed capacity (MW or GW)	Production capacity (MWh or GWh)	Hydraulic head	Flow rate
Anindito et al., 2019	Hydropower reservoir	• Power ➢ Min: 30 MW ➢ Max: 300 MW • BESS: 40 MW (model input)	• Energy storage ➢ RRR: 100 MWh (P8) ➢ BESS: 100 MWh (129 MWh nominal)	Not specified	• Assumed (model input): ➢ MIF: 5 m³/s ➢ MRR: 10-25 m³/s/h
Arvesen et al., 2025	210 HPPs and reservoirs	Not specified	Not specified; 3.0–3.1 TWh/yr reduction due to constraints (~2% of Norway's total)	Not specified	0.05–52.3 m³/s (Environmental flow requirements)
Aubin et al., 2025	Not specified (1288 plants modeled)	32 GW	140–150 TWh/year	Not specified	Minimum flow constraints: 2%, 5%, or 8% of maximum flow at each plant; Maximum ramping: 15–25% of max flow per hour
Bejarano et al., 2019	9 HPPs	Not specified	Not specified	**Gross Head (m):** 1. Arlas: 6.7 2. Etxauri: 10 3. Barazpea: 3.7 4. Bohi: 187.4 5. Pont de Suert: 90.5 6. Bono: 81.7 7. Huérmeda: 4.87 8. Algar de Mesa: 17.3 9. La Requijada: 74	**Maximum turbined** **flow (m** ^**3**^ **/s):** 1. Arlas: 70 2. Etxauri: 15 3. Barazpea: 4 4. Bohi: 11.5 5. Pont de Suert: 21.1 6. Bono: 6 7. Huérmeda: 4.7 8. Algar de Mesa: 1.2 9. La Requijada: 2
Bieri et al., 2016	• Mauvoisin II scheme (project canceled at the end) and Plessur hydropower scheme • Mauvoisin II scheme: 2 power plants: Fionnay and Riddes • Plessur project: 3 HPPs: Litzirüti HPP (upstream), Chur-Sand HPP (downstream), Lüen HPP (in between)	**1. Mauvoisin II scheme** • Rated capacity: Fionnay 128 MW and Riddes 225 MW • Planned power output: from 350 MW to 900 MW 2. Plessur project • Litzirüti HPP: 4.7 MW, Chur-Sand HPP: 8.8 MW, Lüen HPP: 6.7 MW • Planned Pradapunt HPP: 10 MW	Not specified	➢ Mauvoisin II scheme: 1500 m (gross head) ➢ Plessur project: 400 m (planned, net head)	**1. Mauvoisin II scheme** • Rated discharge: ➢ Fionnay: 34.5 m^3^/s ➢ Riddes: 29 m^3^/s for • The maximum release would have risen from 29 m^3^/s to 75 m^3^/s
Casas-Mulet et al., 2016	Lundesokna hydropower	Total installed capacity: 61 MW	Average annual production: 278 GWh	Not specified	0.3 m^3^/s to 19 m^3^/
Casas-Mulet et al., 2014	Lundesokna hydropower	Total installed capacity: 61 MW	Average annual production: 278 GWh	Not specified	From 0.3 m^3^/s (no power production) to 20 m^3^/s (full production)
Chalishazar et al., 2022	Hydropower plant with 4 turbine units	• Per unit: 24.3 MW (total: 97.2 MW) • Annual mileage (MW) ➢ Peaking: 34860 ➢ Run-of-river: 321	Not specified	Not specified	Hydraulic capacity: 6839 ft^3^/s
Dalcin et al., 2024	• Parana River Basin hosting 65 HPPs • Flow regime restoration was analysed at a site between Porto Primavera and Itaipu	• Installed capacity of 65 HPPs: 48,381 MW • Porto Primavera: 1540 MW • Itaipu: 14,000 MW	Not specified	Not specified	Not specified
Gostner et al., 2011	Glurns and Kastelbell	Installed capacity: 90 and 93 MW, respectively	Energy production/year: 291 and 438 GWh, respectively	Net head: 586 and 294 m, respectively	18 m^3^/s and 30 m^3^/s, respectively
Guisández et al., 2013	Dam-based scheme with 3 hydro units	• Maximum power: 319.7 MW (3*106.6) • Minimum power: 21.9 MW	Not specified	• Water elevation (masl): ➢ Maximum: 329.50 ➢ Minimum: 270 ➢ Tailwater: 198	• Maximum flow: 279 m^3^/s (3*93) • Minimum flow: 40 m^3^/s
Guisández et al., 2016	12 hydropower plants	• Power ➢ Maximum: 1.4-316.8 MW ➢ Minimum: 0.2-22.2 MW	Not specified	• Net head (m): ➢ Maximum: 20-259 ➢ Minimum: 11-230	• Maximum plant flow: 8-279 m^3^/s • Minimum plant flow: 2-40 m^3^/s • Maximum outlet flow: 18-432 m^3^/s • Average river flow: 0.6-91.2 m^3^/s
Helseth et al., 2023	Røssåga hydropower system (2 large power stations with upstream reservoirs)	Not specified	Not specified	Not specified	• Minimum discharge constraint: 30 m^3^/s • Ramping constraint: 7.5 m^3^/s per 15 min • Maximum discharge at downstream power station: 160 m^3^/s
Höfkes et al., 2024	3 anonymized HPPs in Switzerland	• Power capacity (MW) ➢ HPP A: 90 ➢ HPP B: 60 ➢ HPP C: 190 • BESS power capacity (MW) ➢ HPP A: 12 ➢ HPP B: 24 ➢ HPP C: 51 • Hybrid (basin+BESS) power capacity (MW): ➢ HPP A: 18 ➢ HPP B: 42 ➢ HPP C: 51	• BESS energy capacity (MWh) ➢ HPP A: 20 ➢ HPP B: 15 ➢ HPP C: 94 • Hybrid (basin+BESS) energy capacity (MWh): ➢ HPP A: 17 ➢ HPP B: 13 ➢ HPP C: 94	Not specified	• Design discharge (m^3^/s) ➢ HPP A: 60 ➢ HPP B: 20 ➢ HPP C: 20 • Max. discharge variation (m^3^/s) ➢ HPP A: 21 ➢ HPP B: 19 ➢ HPP C: 13
Huuki et al., 2022	Taivalkoski hydropower plant	Not specified	Not specified	Head height: 15 m	• Mean flow: 473 m^3^/s • Benchmark operation constraints: ➢ Maximum flow: 726 m³/s ➢ Minimum flow: 101 m³/s ➢ Flow ramping: ±438 m³/s per hour
Jones et al., 2016	Glen Canyon Dam	Generating capacity: 1320 MW	Not specified	Not specified	Not specified
Juutinen et al., 2025	• Ossauskoski HPP • The studied river reach exists between two HPPs, i.e., Ossauskoski (upstream), and Taivalkoski (downstream)	• Ossauskoski: 124 MW (upstream) • Taivalkoski: 134.1 MW (downstream)	The average annual generation is 501 GWh	Not specified	• Annual median hourly ramping: 125- 175 m^3^/s for the past decade • Annual median 200 m^3^/s in summer and overall range 100 to 400 m^3^/s
Kang and Choi, 2019	Goesan Dam	Not specified	Not specified	Not specified	• Average discharges ➢ Drought flow (Q_355_): 1.82 m³/s ➢ Low flow (Q_275_): 4.02 m³/s ➢ Normal flow (Q_185_): 7.23 m³/s ➢ Averaged-wet flow (Q_95_): 17.13 m³/s • Peak discharge: 15 m^3^/s • Peak discharge for Scenario 1 (magnitude - duration concept): 98 m³/s (assumed/modeled data)
Kataria, 2009	• 1900 HPP • 700 large scale plant	700 stations above 1.5 MW capacity	Not specified	Not specified	Not specified
Kern and Characklis, 2017	Roanoke Rapids Dam	100 MW project	The weekly production range based on water availability is 252 MWh to 2000 MWh	Not specified	• Turbine capacity (water release) 20,000 CFS • Minimum release requirement: 325 CFS
Kotchen et al., 2006	2 hydropower dams, Tippy dam and Hodenpyl dam	• Generation capacity: ➢ Tippy dam: 20.1 MW ➢ Hodenpyl dam: 17 MW	• Mean annual generation (1990-2001): ➢ Tippy dam: 59,541.1 MWh/year ➢ Hodenpyl dam: 38,445.8 MWh/year	Not specified	• Mean annual flow (cfy): ➢ Tippy dam: 56,039,472,000 ➢ Hodenpyl dam: 39,577,680,000 • Minimum legal downstream flow (cfs) ➢ Tippy dam: 871 ➢ Hodenpyl dam: 492
Mathisen et al., 2022	Sokna and Aura	Aura: 310 MW and Sokna: 57.9 MW	Aura: 1661 GWh (annual average in reference case), Sokna: 318.9 GWh (annual average in reference case)	Not specified	Not specified
Meile et al., 2011	Multiple high-head storage schemes (5 gauging stations)	Not specified (10 MW ROR plant proposed)	40 GWh/year (proposed multipurpose basin with 10 MW plant)	Not specified	• The cumulative operational discharge: 273 m^3^/s • Cumulated design discharge (m^3^/s) (Table 2): ➢ Brig: 55; Sion: 140.75; Branson: 214.5; Porte du Scex: 273; Vispa: 34 • Mean annual discharge (since 1976) (m^3^/s): ➢ Brig: 43.2; Sion: 109.3; Branson: 142.2; Porte du Scex: 190.8; Vispa: 17.9 • Porte du Scex: ➢ Peak 230 m^3^/s ➢ Minimum daily flow: 100 m^3^/s
Niu and Insley, 2016	Abitibi Canyon generating station	Generation capacity: 336 MW	Not specified	Not specified	• Average hourly inflow rate: 6,671 CFS • Up-ramping and down-ramping constraints: 3000 (CFS-hr) • Water release requirement: ➢ Minimum 2000 CFS ➢ Maximum 15,000 CFS
Niu and Insley, 2013	Abitibi Canyon generating station: Five generating units	• Generation capacity: 0 to 336 MW	Not specified	Not specified	• Water release: 0 (off-peak) to 11,343 CFS (peak)
Olivares and Lund, 2012	Hypothetical hydropower plant	• Installed capacity**:** 25 MW	Not specified	Hydraulic head: 32 m (average head used in tests)	• Turbine flow capacity: 28.3 m³/s • Max. release (per week): 17.1 million m³
Olivares et al., 2015	The main power system in Chile: The Central Interconnected System	• Pangue: 465 MW • El Toro: 450 MW • Machicura: 95 MW	Not specified	Not specified	**1. Pangue** • Environmental flow through turbines: 50 m^3^/s • Minimum technical flow: 35 m^3^/s • Average river flow: 290 m^3^/s (natural regime) • Flow rate change: <40 m³/s/h (natural regime); up to 150 m^3^/s/h (altered regime) **2. Ralco** • Average inflow: 290 m^3^/s (same as Pangue) • Flow rate change: ➢ Natural regime: <40 m³/s/h ➢ Altered regime: up to 270 m^3^/s/h
Olivares et al., 2021	Chilean power system	• Max power: 420 MW • Min power: 32.5 MW	Not specified	Not specified	• Maximum turbine flow: 504 m^3^/s • Minimum turbine flow: 39 m^3^/s • Inflow Range: 78-510 m^3^/s (based on season)
Person et al., 2014	Oberhasli hydropower scheme	Installed capacity: 650 MW	Generated: approximately 1750 GWh (in 2010)	Not specified	Before mitigation: Peak: 63-96 m³/s Off peak: 5–25 m³/s After mitigation: Peak: 41–64 m³/s Off-peak: 9–38 m³/s
Pérez-Díaz and Wilhelmi, 2010	Dam-based scheme; powerhouse at the toe of the dam	• Power: 38.4 MW (3*12.8) • Maximum power: 42 MW (3*14) • Minimum power: 18 MW (3*6)	Not specified	• Water elevation (masl): ➢ Maximum: 290 ➢ Minimum: 275 ➢ Tailwater: 255.10	Flow: 135 m^3^/s (3*45)
Premstaller et al., 2017	Lana HPP in the Valsura River hydro-scheme	• Lana di Sotto HPP (new mitigation plant) ➢ Max power: 8 MW	• Lana di Sotto HPP (new mitigation plant) ➢ Annual generation: 9.2 GWh	• Lana di Sotto HPP (new mitigation plant) ➢ Net hydraulic head: 56 m	• Maximum turbine discharge of Lana HPP: 26.25 m^3^/s • Discharges in the Valsura hydropeaked stretch (based on month): ➢ Minimum: 0.73-2.94 m^3^/s ➢ Maximum: 24.70-27.47 m^3^/s • Pos. ramp rate: 1.90-2.89 m^3^/s/min • Neg. ramp rate: 1.18-2.19 m^3^/s/min • Lana di Sotto HPP (new mitigation plant) ➢ Max flow: 16 m^3^/s
Ruokamo et al., 2024	16 hydropower plants on the main Kemijoki River	Nominal capacity: 1098 MW	• Hydropower production in 2020: 4131 GWh • Assumed/scaled values used for modeling ➢ Hourly hydropower output: minimum 158.6 MWh, maximum ~938.7 MWh ➢ Reservoir storage capacity: from 253 GWh to 1701.2 GWh ➢ Total annual inflow energy: 4350 GWh	Not specified	Not specified
Tonolla et al., 2017	Innertkirchen I and II (operated by Kraftwerke Oberhasli AG (KWO))	Not specified	Additional energy gain: 70 GWh/a	Not specified	• Turbine capacity of Innertkirchen I: ➢ Current: 40 m^3^/s ➢ Planned: 65 m^3^/s • Maximum total flow release in Innertkichen: ➢ Current: 70 m^3^/s ➢ Planned: 95 m^3^/s • In the hydropeaking section: ➢ Mean annual discharge 35 m^3^/s ➢ Natural minimal flow in winter: 2.4 m^3^/s (Q_347_) ➢ During typical floods: 190 m^3^/s (HQ_2_) ➢ Occasional winter floods: reach 40 m^3^/s
